# Avoiding iatrogenic thrombo-embolism: the "KAPLIT" technique

**DOI:** 10.1186/1757-7241-18-53

**Published:** 2010-10-13

**Authors:** Kapil Chaudhary, Lalit Gupta, Raktima Anand

**Affiliations:** 1 Maulana Azad Medical College & associated Lok Nayak Hospital, New Delhi, India

## Abstract

In patients with traumatic injury of an upper limb it is often necessary to both secure intravenous (IV) access and record blood pressure noninvasively in the other upper limb. This may cause intermittent obstruction to the flow of IV fluids during cuff inflation. Also backflow of blood into the IV tubing when the cuff is inflated and the temporary stasis which occurs predisposes to clotting of blood in the IV tubing/catheter. Overenthusiastic efforts to push IV fluids without disconnection and flushing of IV line may pose a possible risk of embolizing the clotted blood thrombus into circulation. We describe a simple technique to prevent backflow of blood into the IV tubing when both intravenous fluid infusion and non-invasive blood pressure cuff are in the same limb. This may prevent clot formation and eliminate the risk of an iatrogenic thrombo-embolism.

## Text

Patients presenting to the emergency department with multiple trauma often require aggressive fluid resuscitation and constant monitoring of their arterial blood pressure. In patients in whom one upper limb is already compromised as a result of trauma both intravenous (IV) fluid infusion and non-invasive blood pressure (NIBP) monitoring have to be done in the other upper limb. IV line placement in the lower limbs is generally avoided because of associated increased risk of thrombophlebitis. Also, the appropriate size thigh cuff for NIBP may not be available especially in the emergency department where such cases often present.

**Figure 1:**
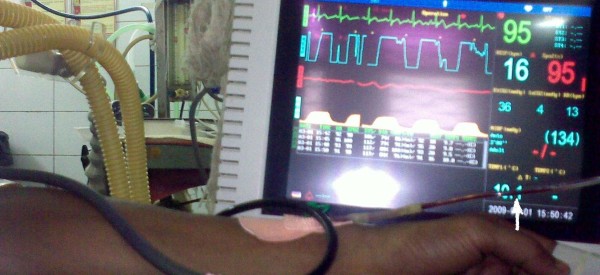
**Back flow of blood in IV tubing**. Back flow of blood (arrow) in IV tubing during NIBP measurement which may lead to clot formation if neglected.

Venous stasis and hypercoagulabilty state have been documented to predispose to thrombus formation (Virchow's triad). Stasis of blood [1] resulting from repeated venous occlusion and back flow of blood into the IV tubing [2] with cuff inflation during NIBP measurement (Figure 1) may lead to occlusion of the IV catheter/tubing from thrombus formation, especially if the NIBP measurement interval is short or the IV line is left unnoticed for some time. Intraluminal clot formation accounts for 5-25% of all catheter occlusions [3]. This requires disconnection and flushing of IV line which poses a risk of catheter infection with repeated handling and further predisposing to thrombus formation [3]. Enthusiastic efforts by beginner resident doctors or technical support staff to restore IV line patency, without disconnection and flushing of line, by compressing the IV tubing/fluid vac (to apply forward positive pressure) may lead to embolization of this clot into the circulation. Pulmonary embolism has been noted in 16% patients with catheter related thrombosis (13% non-fatal and 3% fatal) [4,5]. This may be of real concern especially in patients with heart disease, cerebrovascular disease and in prothrombotic states. Moreover, general anaesthesia too is a prothrombotic state and such patients undergoing surgery may be at an additional risk for thrombo-embolism.

**Figure 2:**
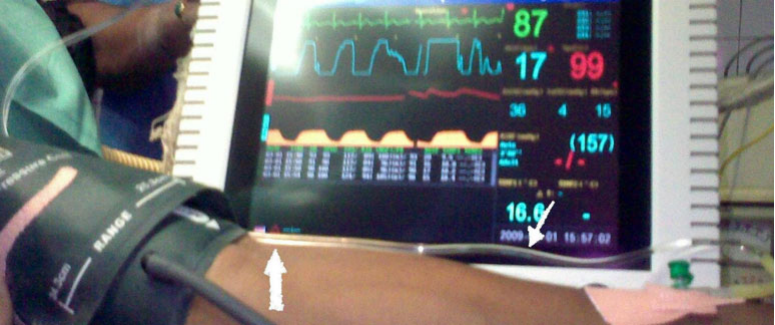
**Avoiding back flow using "KAPLIT" technique**. No back flow of blood (thin arrow) in IV tubing during NIBP measurement when tubing passed between the NIBP cuff beneath the artery mark and upper arm (bold arrow): the "KAPLIT" technique.

We have found that if the IV tubing is passed between the NIBP cuff and upper arm (the "KAPLIT" technique) it gets compressed whenever the cuff inflates to measure BP (Figure 2). This is similar to manually closing the IV line each time BP is measured. This simple, easy and non-time consuming technique which does not require any additional equipment or manpower obviates the need for repeated manual closure or flushing of the IV line along with preventing any backflow of blood/venous stasis (Figure 2) and resultant thrombus formation. The prevention of catheter/tubing occlusion thus eliminates the need for applying positive pressure to restore IV line patency and clot embolization. Also it benefits the anaesthesiologist in the operation room to monitor NIBP at frequent intervals without constant supervision of the IV line and interruption of fluid resuscitation.

NIBP monitoring has long been considered to be a safe monitoring method. Underreporting of the complications associated with NIBP monitoring has lead to limited awareness among clinicians of its potential complications [1]. The reported complications with its use include petechial rash, ecchymoses, skin necrosis, infection, thrombophlebitis, venous stasis, compressive neuropathy and compartment syndrome [1]. Although the incidence of iatrogenic thrombo-embolism resulting from embolization of clot formed in IV tubing due to venous stasis with NIBP measurement has not been reported, the potential risk still exists as described. This risk of iatrogenic thrombo-embolism may be very small, but its prevention cannot be over-emphasized when compared to the morbidity and mortality when such a complication occurs apart from the time and resources consumed especially when a simple technique (the "KAPLIT" technique) can be used.

## Abbreviations

IV: intravenous; NIBP: non invasive blood pressure.

## Competing interests

The authors declare that they have no competing interests.

## Authors' contributions

KC conceived of the technique and participated in its design and coordination, observing the efficacy and drafted the manuscript. LG helped in observing the efficacy of technique and preparation of manuscript. RA helped to draft the manuscript and gave final approval to submit manuscript. All authors have read and approved the final manuscript.

## Author's information

KC- Senior Resident, Department Of Anaesthesia and Intensive Care, Maulana Azad Medical College & associated Lok Nayak Hospital, New Delhi, India

LG- Ex-DNB Student, Department Of Anaesthesia and Intensive Care, Maulana Azad Medical College & associated Lok Nayak Hospital, New Delhi, India

RA- Director, Professor and Head, Department Of Anaesthesia and Intensive Care, Maulana Azad Medical College & associated Lok Nayak Hospital, New Delhi, India
